# Temporal succession and assembly of marine bacterial communities in Maxwell Bay, Antarctica during summer

**DOI:** 10.3389/fmicb.2026.1748960

**Published:** 2026-03-19

**Authors:** Haiyu Zeng, Zhiwei Gao, Zheng Wang, Kaiyi Li, Bo Xu, Zhen Yan, Yan Gu, Weimeng Du, Haitao Ding, Jianjun Wang

**Affiliations:** 1 School of Ecology, Hainan University, Haikou, China; 2 Antarctic Great Wall Ecology National Observation and Research Station, Polar Research Institute of China, Ministry of Natural Resources, Shanghai, China; 3 Key Laboratory for Polar Science, Polar Research Institute of China, Ministry of Natural Resources, Shanghai, China; 4 State Key Laboratory of Marine Resource Utilization in South China Sea, Hainan University, Haikou, China; 5 School of Oceanography, Shanghai Jiao Tong University, Shanghai, China; 6 School of Marine Science and Engineering, Hainan University, Haikou, China

**Keywords:** Antarctic microbiome, community assembly, environmental selection, null model, temporal succession

## Abstract

**Introduction:**

In recent years, ecological feedbacks driven by climate change have become increasingly prominent. The polar amplification effect has made Antarctic ecosystems pivotal indicators for reflecting global climatic impacts. As core drivers of biogeochemical cycling, marine microbes play a central role. Therefore, deciphering their temporal dynamics and assembly mechanisms is crucial for projecting the trajectories of polar ecosystems. However, the intrinsic ecological processes regulating microbial summer succession, particularly the relative contribution of deterministic processes, remain insufficiently quantified.

**Methods:**

In the present study, Maxwell Bay, Antarctica—a coastal marine region heavily influenced by glacial melt—was selected as the model system. Surface seawater samples were collected sequentially during the 2022 austral summer, followed by 16S rRNA gene amplicon sequencing and phylogenetic null model analysis.

**Results:**

Our results revealed a distinct shift in the assembly mechanisms of bacterial communities. In January, community structure was shaped jointly by stochastic and deterministic processes, with stochastic processes contributing a greater proportion to assembly. This state transitioned to the predominance of deterministic homogeneous selection (84.68%) in February. Mantel tests, followed by linear regression analyses, confirmed that this phylogenetic transition was driven by shifting environmental factors. Specifically, water temperature served as the primary influencing factor in January, whereas silicate and nitrate concentrations emerged as the key factors in February. Subsequent partial least squares path modeling (PLS-PM) and redundancy analysis (RDA) further validated these findings, demonstrating that the identified environmental variables collectively explained more than 50% of the observed variation in community structure. Notably, nitrate drawdown was significantly correlated with the increased relative abundances of dominant bacterial genera in February.

**Discussion:**

By quantifying the relative roles of deterministic and stochastic processes in microbial community assembly, this study demonstrates that environmental selection is the dominant factor mediating microbial responses to polar warming. These findings provide a mechanistic foundation for the development of predictive models for future marine biogeochemical cycles in polar regions.

## 1 Introduction

Climate change exerts multidimensional threats to global ecosystems, encompassing increasingly frequent extreme weather events, rising sea levels, and declining ecosystem stability (Mora et al., 2018). In this context, Antarctica has emerged as both a hotspot and an early-warning hub for global change, amplifying by the polar amplification effect, a phenomenon wherein polar regions undergo significantly faster warming than the global average, driven by intricate feedback mechanisms. As a key regulator of global climate and biogeochemical cycling, the Southern Ocean not only serves as a major carbon sink (Henley et al., 2020; Isla, 2022), but also exerts profound influence on global nutrient dynamics through its unique high-nutrient and low-chlorophyll characteristics (Convey et al., 2009; Wilkins et al., 2013; Murphy et al., 2021). Notably, this region is undergoing rapid changes, including accelerated sea-ice retreat, increased glacial meltwater input, and shifts in species composition (Barbraud et al., 2012; Jarnikova et al., 2025; Nack et al., 2025). These processes are projected to trigger large-scale feedback effects through physical circulation and the biological pump (Davidson et al., 2016; Ong et al., 2025), underscoring the urgent need to clarify the response mechanisms of ecosystems in this region.

Microorganisms are pivotal drivers of marine biogeochemical cycling of carbon, nitrogen, and sulfur, mediating these processes via both phototrophic and heterotrophic metabolisms (Cavicchioli, 2015; Steinberg et al., 2017). They also underpin organic matter degradation and nutrient regeneration, in turn sustaining the energy and material fluxes of marine ecosystems (Wilkins et al., 2013). As key regulators of Antarctic ecosystems, marine microbial communities are shaped by multiple environmental factors while exhibiting distinct temporal dynamics and ecological adaptability (Piedade et al., 2024; Faria et al., 2025; Zhang S. S. et al., 2025; Zhang Y. H. et al., 2025). Owing to their rapid responsiveness to environmental perturbations, these microbial communities serve as sensitive early indicators of ecosystem responses to global change. Shifts in their composition and structure not only provide valuable insights into environmental disturbances, but also lay a foundation for projecting ecosystem trajectories (Goldenberg-Vilar et al., 2025; Iqbal et al., 2025). Current research on Antarctic microbial communities has focused heavily on spatial heterogeneity. Microbial assemblages vary markedly across different habitats. For instance, ice and snow habitats support specialized psychrophilic bacterial taxa adapted to extreme cold and oligotrophic conditions, whereas soils and seawater are predominantly characterized by terrestrial-derived and marine-specific bacterial lineages, respectively (Bendia et al., 2023).

However, relative to spatial patterns, research on short-term temporal dynamics and community succession remains inadequate, particularly over successive austral summers. Characterized by rapid environmental shifts and peak biological activity, the austral summer presents a critical window to explore microbial responses (Luria et al., 2017; Rozema et al., 2017; Siman-Tov, 2022). Maxwell Bay, located in the Southern Ocean and adjacent to the ice-free, biodiverse King George Island, serves as a microcosm of Antarctic microbial communities (Rego et al., 2020; Bello et al., 2022). This distinctive setting provides as an ideal natural laboratory for exploring the adaptability and resilience of microbiome in extreme environments (Zhang et al., 2018). Conducting temporally resolved analyses of microbial succession, community assembly processes, and associated environmental drivers in such system is essential to advancing our understanding of the adaptation and resilience maintenance of polar ecosystems.

**Figure 1:**
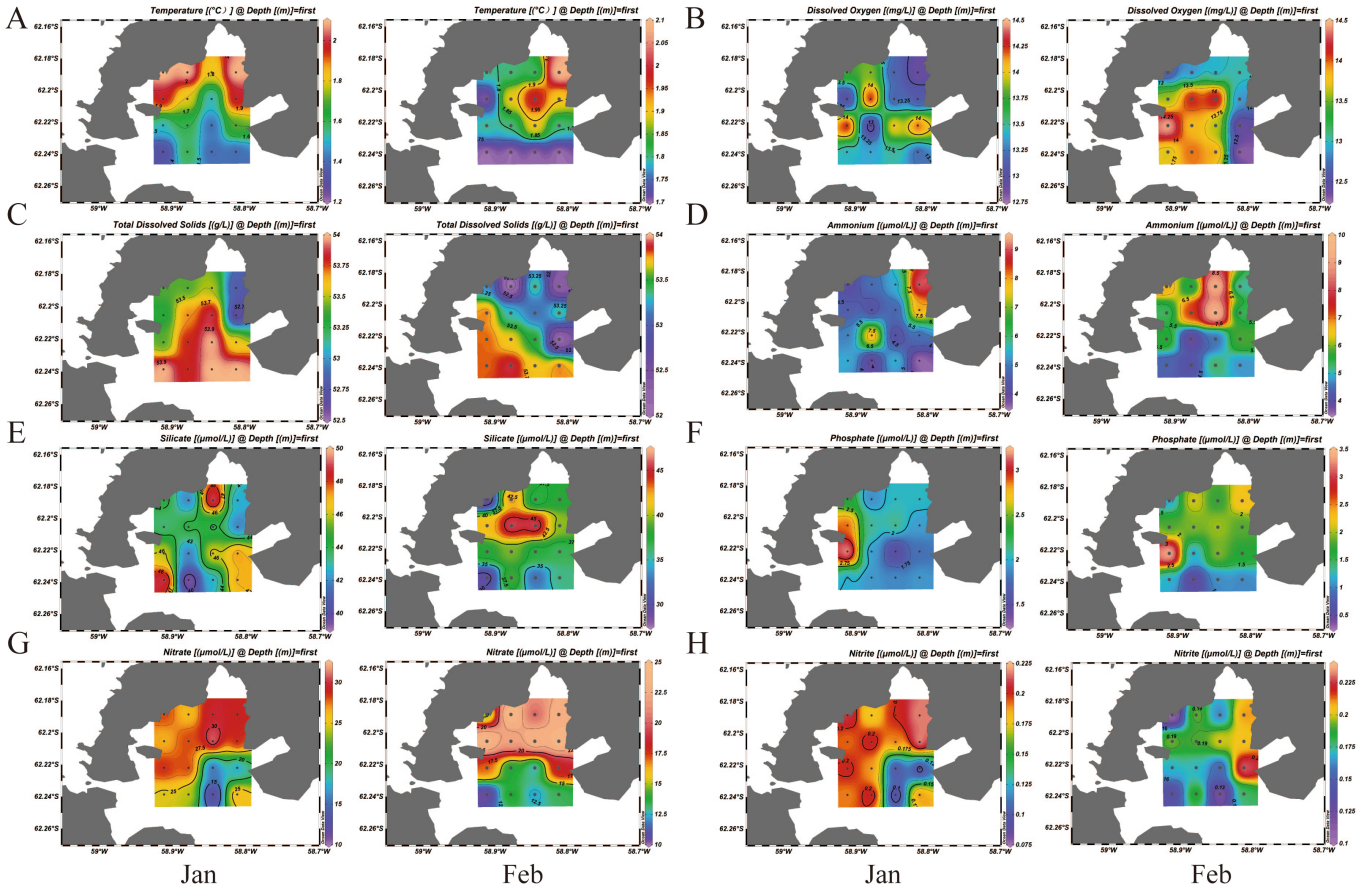
Map showing the sampling sites information (Jan and Feb). **(A)** Temperature of samples. **(B)** Dissolved oxygen of samples. **(C)** Total dissolved solids of samples. **(D)** Ammonium concentration of samples. **(E)** Silicate concentration of the samples. **(F)** Phosphate concentration of the samples. **(G)** Nitrate concentration of the samples. **(H)** Nitrite concentration of the samples.

Previous studies have explored microbial communities in regions adjacent to Maxwell Bay, including effluent zones near research stations and Marian Cove (Zeng et al., 2014; Amaro et al., 2015; Luo et al., 2016; Kim et al., 2020; Liu and Jiang, 2020). Separately, Wang et al. (2024) characterized the composition and potential functions of prokaryotic and eukaryotic communities in specific regions of the bay during January. However, comprehensive surveys covering the entire bay during the interstage transition period of summer remain limited. Notably, the temporal succession of microbial communities and associated shifts in assembly mechanisms during the peak summer melting period remain incompletely understood. To address these knowledge gaps, we conducted a detailed comparative investigation across 16 sampling sites in Maxwell Bay from January to February 2022. This time window is ecological significant, as it coincides with the pronounced environmental transition characteristic of the South Shetland Islands. Focusing on the physicochemical gradients characteristic of this period, evidenced by significant depletion of inorganic nutrients and fluctuations in water temperature (Supplementary Table S1 and Figure 1), this study aimed to: (1) characterize the successional dynamics of prokaryotic community during this dynamic stage; and (2) To evaluate shifts in the relative contributions of stochastic and deterministic assembly processes, we further identified how environmentally driven temporal variations regulate microbial successional trajectories during the summer. Our findings offer new insights into the mechanisms underlying polar microbial responses to climate change and lay a theoretical basis for predicting the future development trends of Antarctic marine ecosystems.

## 2 Materials and methods

### 2.1 Study area and sample collection

**Figure 2:**
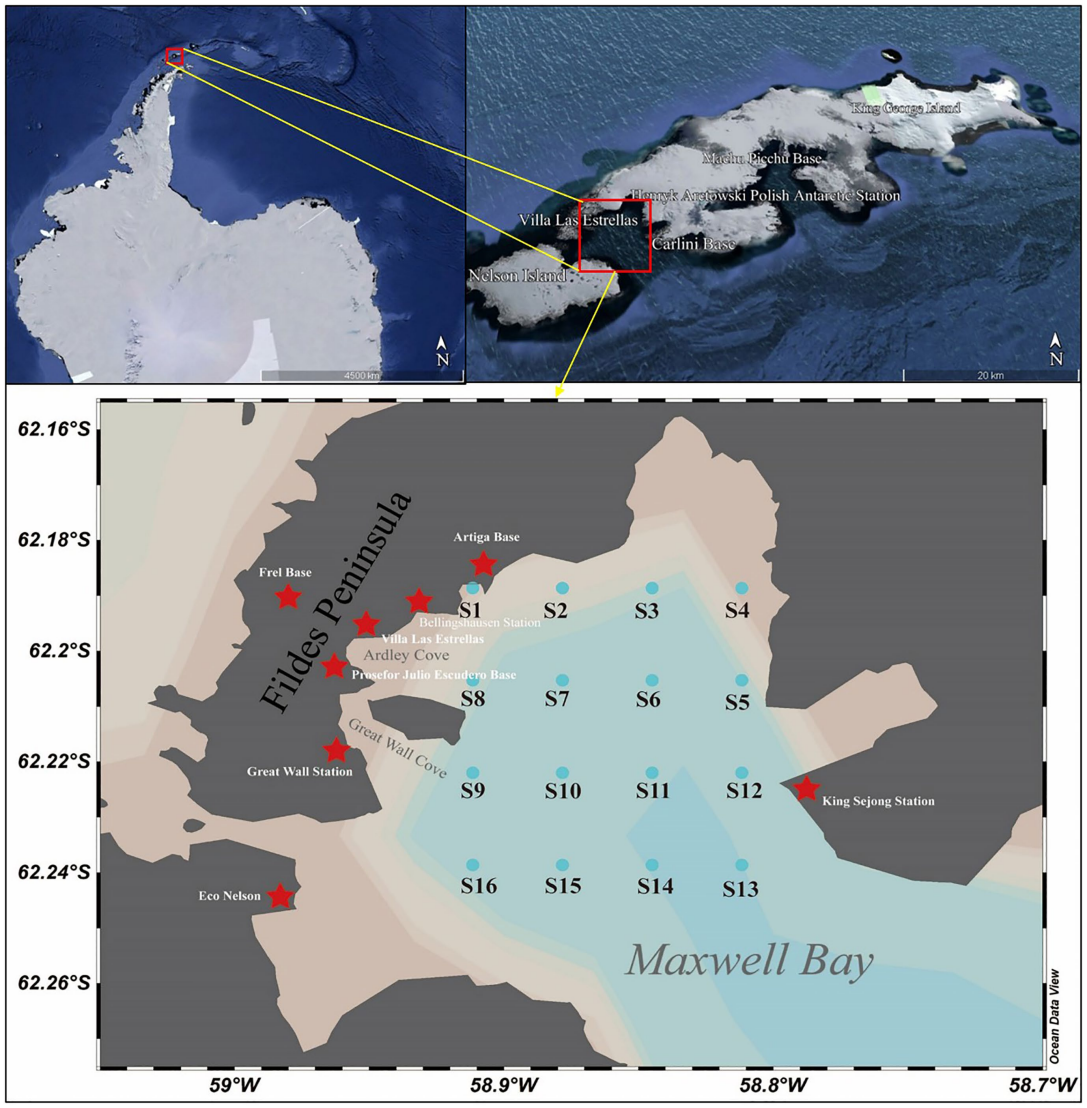
Sampling location map of Maxwell Bay, Antarctica. Cyan dots denote the 16 sampling sites (S1–S16), and red stars mark the scientific research stations along the Fildes Peninsula. Geographic coordinates (latitude and longitude) are shown on the axes for precise spatial referencing, with the vertical axis oriented northward. Grid lines on the latitude axis are spaced at an approximate interval of 2.2 km. Inset maps illustrate the location of the study area within King George Island and the wider Antarctic region.

All seawater samples were collected during China’s 38th Antarctic Research Expedition, conducted from January to February 2022 (austral summer). Surface seawater (0–5 m depth) was collected using a Niskin bottle at 16 sampling sites across Maxwell Bay, King George Island, Antarctica. These sites cover geographical coordinates ranging from 62°11′19″S to 62°14′19″S and 58°48′42″W to 58°54′42″W (Figure 2). At each site, 1 liter of seawater was pre-filtered through a 20-μm mesh to remove larger zooplankton and particulates, followed by filtration through a 0.2-μm pore-size polycarbonate membrane filter (Whatman, United Kingdom). All filters were immediately stored at −80° C pending further laboratory processing.

### 2.2 Environmental parameter measurements

Key physicochemical parameters water temperature, pH, dissolved oxygen (DO), and total dissolved solids (TDS) were measured on-site with a Multi3630S water quality multi-parameter analyzer (WTW, Germany). For subsequent nutrient analysis, water samples were immediately filtered through 0.45 μm mixed cellulose ester (MCE) membranes (Whatman, United Kingdom). The filtrate was then preserved by freezing at −20 °C pending laboratory analysis. Concentrations of dissolved inorganic nutrients were quantified using a SmartChem 450 automated chemical analyzer (AMS Alliance, Italy), following spectrophotometric protocols specific to ammonium (US EPA Method 350.1), nitrite (Bendschneider and Robinson, 1952), nitrate (Morris and Riley, 1963), phosphate (Murphy and Riley, 1962), and silicate (Thomsen et al., 1983) respectively.

### 2.3 DNA extraction, qualification, and sequencing

Total microbial genomic DNA was extracted from the filters using the E. Z. N. A.™ MagBind Soil DNA Kit (Omega, M5635-02, United States). DNA concentration was measured with a Qubit 4.0 Fluorometer (Thermo Fisher Scientific, United States) to verify sufficient yields of high-quality genomic DNA. The V3–V4 hypervariable region of the bacterial 16S rRNA gene was amplified with primers 338F (5’-ACTCCTACGGGAGGCAGCAG-3′) and 806R (5’-GACTACHVGGGTWTCTAAT-3′) (Caporaso et al., 2011), using 2 × Hieff^®^ Robust PCR Master Mix (Yeasen, China). Purification of the amplified products was performed with Hieff NGS™ DNA Selection Beads (Yeasen, China) to remove free primers and primer dimers. The DNA concentration of each amplicon was assessed using an Agilent 2,100 Bioanalyzer (Agilent Technologies, United States). Purified amplicons were used for library preparation, incorporating Illumina adapters and dual-index barcoding. Paired-end sequencing (2 × 300 bp) was conducted on an Illumina MiSeq platform (Illumina, USA) at Sangon Biotech (Shanghai, China).

### 2.4 Analysis of microbial community

Following sequencing, paired-end reads were merged with PEAR software v0.9.8 (Zhang et al., 2014) based on overlap consensus, producing FASTA files and quality data for downstream analysis. High-quality sequences were clustered into operational taxonomic units (OTUs) at a 97% similarity cutoff using Usearch 11.0.667 (Edgar, 2013; Edgar, 2016). Chimeric sequences and singleton OTUs (those represented by a single read) were filtered out. The remaining sequences were mapped to samples according to OTU membership, and the most abundant sequence in each cluster was chosen as the representative sequence. Taxonomic assignment of prokaryotic OTUs was conducted against the Silva v138.2 database (Quast et al., 2012). To preserve the biological integrity of prokaryotic community profiles, sequences annotated as mitochondria or chloroplasts were rigorously filtered out. The resulting OTU table was then rarefied to a uniform minimum depth of 60,000 reads per sample, ensuring unbiased comparisons of diversity and community structure across all sampling sites.

### 2.5 Statistical analyses

All statistical analyses were conducted in R 4.4.3 to characterize microbial diversity patterns, disentangle community assembly mechanisms, and explore environmental interactions. Alpha and beta diversity metrics were calculated using the vegan package 2.7.1. Permutational multivariate analysis of variance (PERMANOVA) with 999 permutations was used to test for significant differences in community structure. The phylogenetic null model approach, which incorporates the Beta Nearest Taxon Index βNTI and Raup-Crick metric *RC_bray_*, was employed to quantify the relative contributions of stochastic and deterministic processes to community assembly. To rigorously quantify the environmental drivers and minimize potential biases arising from composite variables, individual environmental variables were standardized using *Z*-score normalization, and their individual effects on the βNTI matrix were evaluated independently through Mantel tests and linear regression analyses. Redundancy Analysis (RDA) was further used to evaluate the effects of environmental factors, with the statistical significance of each variable determined via Monte Carlo permutation tests (999 permutations) using the envfit function. Partial Least Squares Path Modeling (PLS-PM) was applied to assess causal relationships among spatial distance, environmental variables, and community structure. Goodness of Fit (GOF) was employed to evaluate the predictive performance and overall reliability of the model. Spearman correlation analysis was used to explore associations between bacterial taxa and environmental factors. To mitigate Type I error risk associated with multiple comparisons, all *p*-values were adjusted via the False Discovery Rate (FDR) method (Benjamini-Hochberg procedure). All visualizations were generated using OriginPro 2025b and R packages based on ggplot2 3.5.2.

## 3 Results

### 3.1 Variations in physicochemical parameters of seawater

Systematic monitoring of surface seawater across 16 sampling sites in an Antarctic bay, conducted during the January–February period, revealed distinct spatiotemporal variability in nutrient concentrations. This pattern reflects intense summer biogeochemical cycling in the coastal zone, despite the relative stability of the seawater’s basic physical properties. Over the entire study period (January–February), all 16 sampling sites showed characteristics typical of Antarctic summer conditions. Basic physical parameters remained stable: mean water temperature was low (1.76 ± 0.03 °C), salinity was high (with total dissolved solids, TDS, reaching 53.44 ± 0.06 g/L), pH was weakly alkaline (7.83 ± 0.01), and dissolved oxygen (DO) levels were sufficient (13.45 ± 0.07 mg/L). In sharp contrast, the concentrations of major nutrients exhibited striking spatiotemporal heterogeneity (Figure 1 and Supplementary Table S1). Temporally, most nutrients followed a consistent depletion trend from January to February (Supplementary Figure S1), which indicates intense biological uptake during summer. Mean nitrate concentration decreased significantly from 25.30 ± 5.70 μmol/L to 18.03 ± 5.21 μmol/L (*p* < 0.01, *t*-test). Silicate depletion was even more notable, declining from 44.41 ± 2.88 μmol/L to 37.94 ± 4.94 μmol/L (*p* < 0.001). Phosphate also declined, though to a lesser degree (from 2.09 ± 0.42 μmol/L to 1.67 ± 0.69 μmol/L). Conversely, the mean ammonium concentration increased slightly, from 5.33 ± 1.67 μmol/L to 5.78 ± 1.71 μmol/L, which suggests that active nitrogen regeneration processes were occurring. Spatially, the nutrient distributions showed clear geographic variability (Figure 1). Nitrate depletion followed a distinct spatial gradient: in January, higher concentrations (>28 μmol/L) were mainly detected at northern sites (S1–S10), while southern sites (S11–S16) had relatively lower levels (15.00–25.00 μmol/L). By February, concentrations had decreased across all sites, with severe depletion concentrated at the southernmost locations (S13–S16, <14 μmol/L), which indicates strong demand for bioavailable nitrogen in this area. The spatial pattern of silicate closely matched that of nitrate: the appearance of distinct low-value zones (<36 μmol/L) at southern sites (S11–S16) in February overlapped spatially with the regions of nitrate depletion, which strongly suggests the occurrence of a significant diatom bloom event. In contrast, ammonium showed a distinctly localized distribution, featuring hotspots (>9 μmol/L) at specific sites such as S3 and S6. These anomalies are likely linked to excretion from nearby penguin colonies or regenerated nitrogen produced by the degradation of local organic matter.

In summary, the results clearly outline the spatiotemporal dynamics of summer nutrient regimes in Maxwell Bay, Antarctica. The depletion of nitrate and silicate, with heightened consumption observed at southern sampling sites, indicates intense primary production activity in this region. At the same time, the localized accumulation of ammonium reflects the combined impacts of organic matter input and nitrogen regenerative processes. These well-defined spatial and temporal variations in nutrient availability offer critical environmental context for interpreting microbial community succession patterns in the study area.

### 3.2 Dynamics of bacterial community characteristics

**Figure 3:**
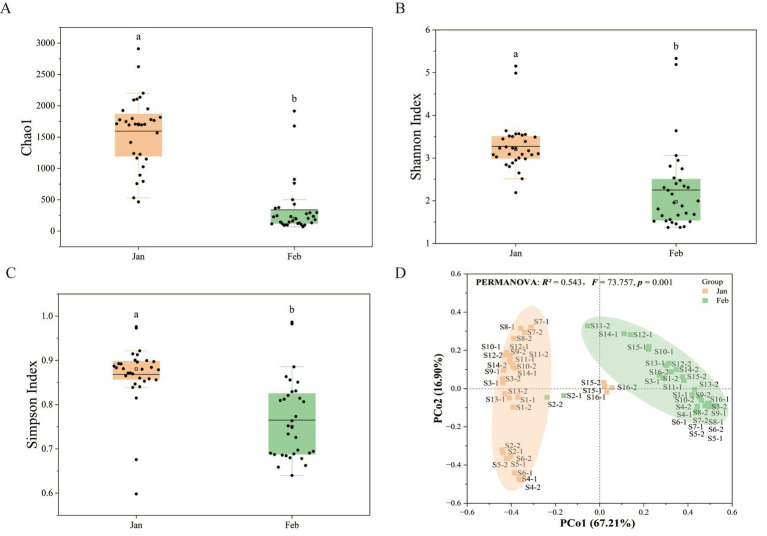
Bacterial community diversity analysis (Jan and Feb). **(A)** Chao 1 by Month. **(B)** Shannon Index by Month. **(C)** Simpson Index by Month. Horizontal lines indicate the median, and lowercase letters above the bars denote significant differences (*p* < 0.05) between the two groups, as determined by Fisher’s exact test. **(D)** Principal coordinates analysis (PCoA) of bacterial community structure based on PERMANOVA analysis.

To characterize the dynamic temporal shifts of bacterial communities during the Antarctic summer, systematic analysis was conducted on bacterial community diversity, structural features, and taxonomic composition. Results from this analysis revealed a significant and consistent pattern of temporal succession in the bacterial community over the period from January to February. Bacterial alpha diversity exhibited a pronounced temporal decline, with all richness and evenness metrics including the Shannon, Chao1, and Simpson indices decreasing significantly from January to February (*p* < 0.01; Figures 3A–C). This pattern indicates a substantial reduction in species diversity and community complexity over the study period. Principal Coordinates Analysis (PCoA) further confirmed a distinct temporal shift in community structure (Figure 3D). The first two axes (PCoA1 and PCoA2) collectively explained 78.11% of the total variation (PCoA1: 61.21%; PCoA2: 16.90%), with samples forming clear monthly clusters. PERMANOVA validated this observation (*R^2^* = 0.54, *p* = 0.001), identifying temporal variation as the primary driver of community dissimilarity, accounting for 54.30% of the observed variance.

**Figure 4:**
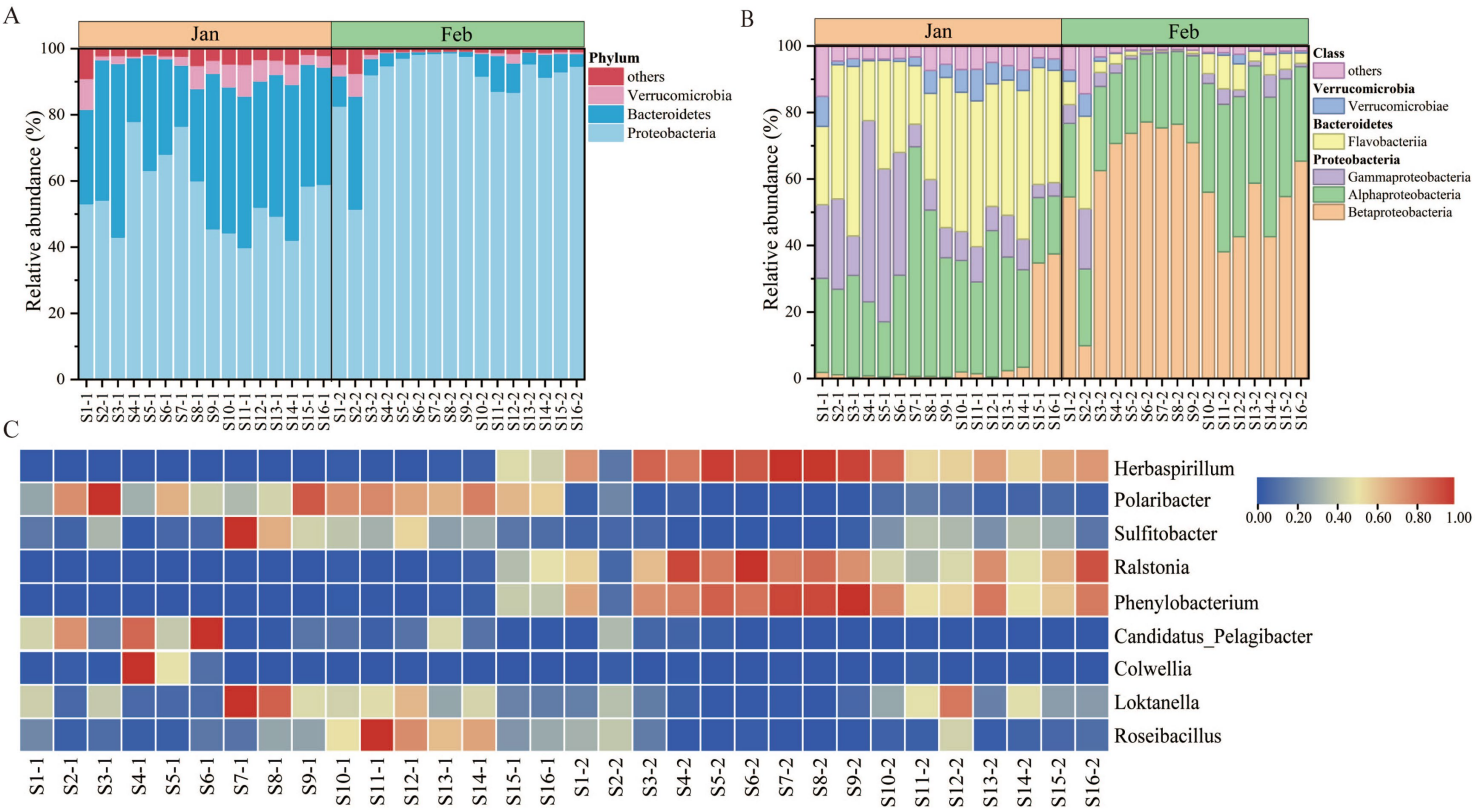
Composition of bacterial communities in January and February. **(A)** Phyla-level composition. This panel displays the composition of bacterial communities at the phyla level with relative abundances greater than 1% in January and February. Phyla with less than 1% abundance are combined and shown in the “Others” category. **(B)** Class-level composition. This panel shows the composition of bacterial communities at the class level with relative abundances greater than 1% in January and February. **(C)** Genus-level composition. This heatmap illustrates the composition of bacterial communities at the genus level with relative abundances greater than 1% in January and February. The color scale represents min-max standardized relative abundance values, ranging from 0.00 (blue) to 1.00 (red).

Taxonomic analysis revealed substantial compositional turnover between the 2 months. At the phylum level, *Proteobacteria* remained the dominant group but underwent marked internal restructuring (Figure 4A). The January community was characterized by a diverse assemblage of *Proteobacteria*, *Bacteroidota*, and *Verrucomicrobiota*, whereas *Proteobacteria* strengthened its dominance by February, with the relative abundances of the latter two phyla declining significantly. This taxonomic restructuring was more prominent at the class level (Figure 4B): the January community, a multi-taxa assemblage dominated by *Alphaproteobacteria* and *Flavobacteriia*, transitioned to a highly specialized structure in February. During this period, *Betaproteobacteria* emerged as the near-exclusive dominant class, substantially displacing other previously abundant taxa.

At the genus level, a distinct successional pattern was observed. The January bacterial community was dominated by representative genera, including *Polaribacter*, *Sulfitobacter*, *Candidatus_Pelagibacter*, *Colwellia*, and *Loktanella*, with moderate spatial variability across sampling sites (Figure 4C). By February, a pronounced shift had occurred: genera such as *Ralstonia*, *Phenylobacterium*, *Herbaspirillum*, and *Roseibacillus* became significantly more prominent and exhibited consistent distribution across most sampling sites (S3–S10, S13, S15–S16). Statistical analyses confirmed that these taxonomic shifts were closely associated with the concurrent nutrient dynamics observed earlier (Section 3.1), specifically the depletion of nitrate and accumulation of ammonium.

### 3.3 Temporal dynamics of bacterial community assembly mechanisms

**Figure 5:**
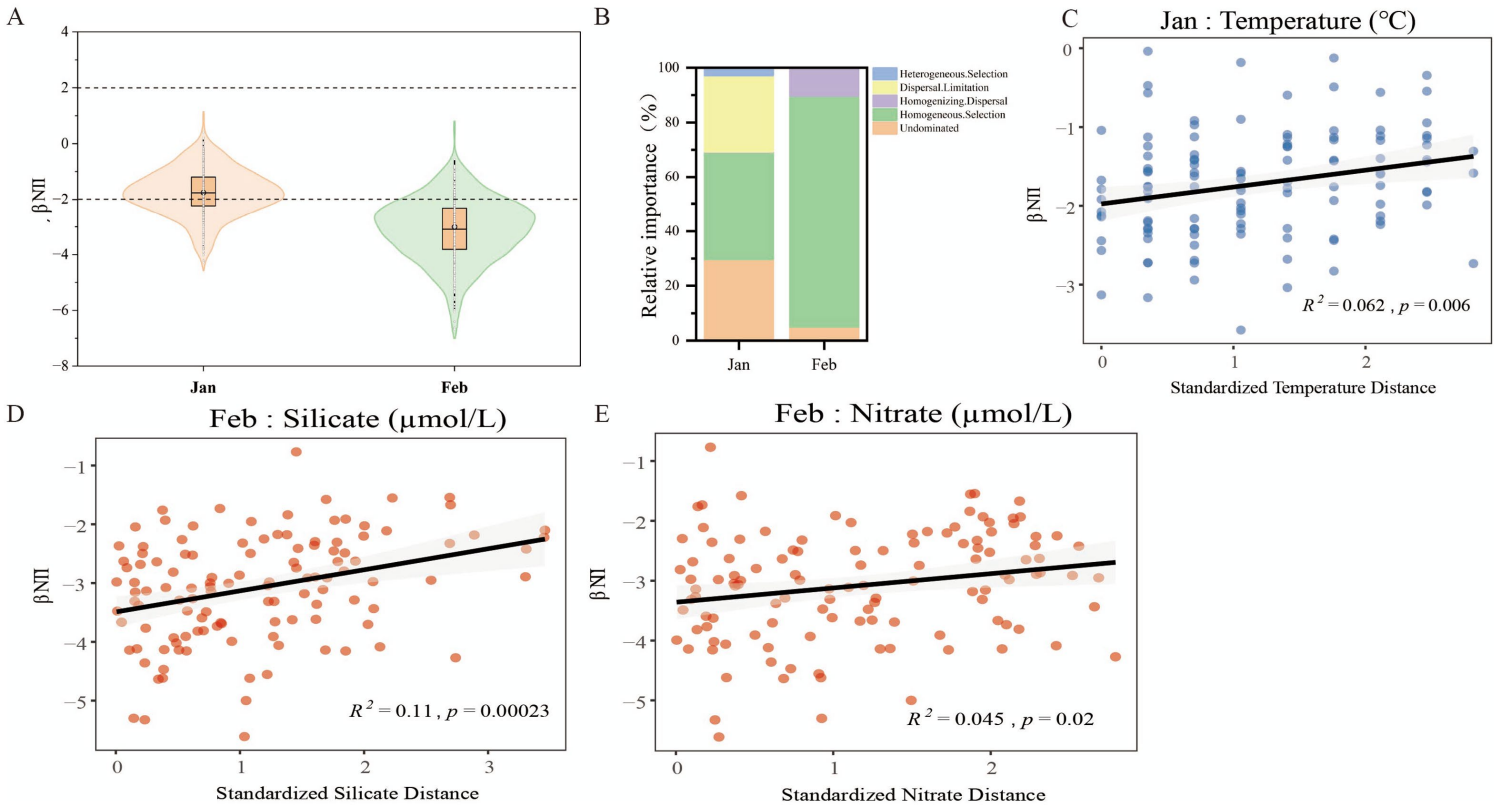
Ecological processes and environmental drivers governing bacterial community assembly in January and February. **(A)** Distribution of the beta nearest taxon index (βNTI) for bacterial communities. Horizontal dashed lines at ±2 represent the significance thresholds for deterministic (βNTI > 2) versus stochastic (βNTI < 2) assembly. **(B)** Relative importance of community assembly pathways partitioned using the βNTI and *RC_bray_* null-model framework. **(C–E)** Identification of key environmental drivers via individual Mantel tests and linear regressions: correlation between βNTI and **(C)** Temperature in January, **(D)** Silicate in February, and **(E)** Nitrate in February. Solid lines represent best-fit linear regressions, and shaded areas denote 95% confidence intervals. *R^2^* and *p*-values are based on standardized environmental distances. Comprehensive statistics for all tested variables are provided in Supplementary Tables S2, S3, with non-significant correlations presented in Supplementary Figures S2, S3.

To further elucidate the distribution patterns and assembly mechanisms of the bacterial community, we applied the phylogenetic null model framework based on the Beta Nearest Taxon Index (βNTI) and Raup-Crick metric (*RC_bray_*). Our results revealed a distinct temporal shift in community assembly: in January, the community structure was shaped jointly by stochastic and deterministic processes, with stochasticity contributing a higher proportion to assembly. Specifically, βNTI values in January were distributed across both stochastic and deterministic ranges (Figure 5A), with the majority concentrated within the [−2, 2] interval. Quantitative partitioning further confirmed this pattern, showing that the combined stochastic component which included dispersal limitation (27.82%) and undominated processes (29.63%) accounted for a relatively higher proportion (57.45%) of total assembly processes. In contrast, homogeneous selection and heterogeneous selection accounted for the remaining 39.51 and 3.04%, respectively (Figure 5B). By February, however, the assembly mechanism underwent a significant transition: βNTI values shifted downward and became more clustered, with the vast majority falling below the −2 threshold (Figure 5A). Correspondingly, the relative contribution of homogeneous selection surged to 84.68%, while the influence of stochastic processes declined markedly.

To identify the key environmental variables influencing bacterial community assembly, we initially employed Mantel tests to evaluate the individual effects of all standardized environmental factors (Supplementary Table S2). Specifically, this initial analysis showed that water temperature was significantly correlated with βNTI in January, whereas silicate and nitrate concentrations emerged as the primary drivers in February (Supplementary Table S2). To build on these findings, linear regression analyses were further performed to quantify the impact of environmental pressures on phylogenetic turnover (Supplementary Table S3). The results demonstrated that the drivers governing deterministic assembly underwent a distinct temporal transition during the summer: in January, water temperature showed a significant positive correlation with βNTI (*R^2^* = 0.062, *p* = 0.006; Figure 5C), with the linear relationships between other environmental factors and βNTI for this month detailed in Supplementary Figure S2. In contrast, community assembly in February was predominantly regulated by nutrient availability; specifically, βNTI values exhibited significant linear correlations with standardized silicate distance (*R^2^* = 0.11, *p* = 0.00023; Figure 5D) and nitrate distance (*R^2^* = 0.045, *p* = 0.02; Figure 5E). The corresponding correlations for other environmental factors in February are presented in Supplementary Figure S3. Collectively, these results indicate that the intensification of deterministic homogeneous selection in late summer is closely linked to temporal fluctuations in temperature and nutrient concentrations within the bay.

### 3.4 Environmental drivers of bacterial community structure

**Figure 6:**
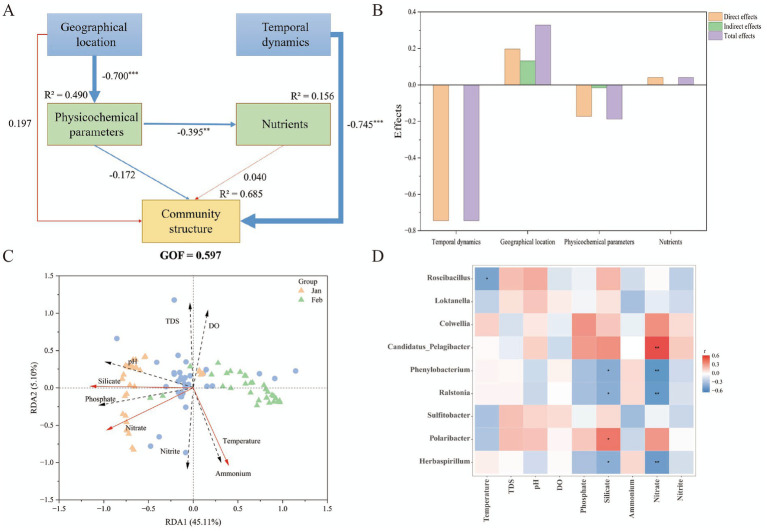
Factors influencing bacterial community structure. **(A)** Partial least squares path modeling (PLS-PM) analysis. This model depicts causal relationships among geographical location (latitude and longitude), temporal dynamics, physicochemical parameters [temperature, total dissolved solids (TDS), dissolved oxygen (DO), and pH], and nutrients [phosphate, silicate, ammonium, nitrate, and nitrite] with respect to community structure. **(B)** Factor contribution. **(C)** Redundancy analysis (RDA) of environmental factors. Blue dots denote microbial genera; triangles represent sampling sites (January: orange; February: green). Red arrows indicate the most significant environmental drivers (*p* < 0.001, validated via 999 Monte Carlo permutations using the envfit function), while black dashed/solid arrows represent other variables. **(D)** Correlation heatmap. Associations between dominant microbial genera (>1%) and environmental factors. Significance levels for panels **(A,D)** are denoted by asterisks: ***p < 0.001, **p < 0.01, *p < 0.05.

To systematically identify the dominant factors and pathways driving shifts in microbial community structure in the Antarctic bay between January and February, we employed a multi-faceted analytical approach, integrating PLS-PM, RDA, and correlation analysis. This integration enabled us to investigate environmental control mechanisms from multiple perspectives, including direct effects, explanatory power, and species–environment associations. Analysis leveraging PLS-PM (Figures 6A,B) revealed temporal variation as the primary direct driver of shifts in community structure, with a pronounced negative direct effect (Estimate = −0.745, *p* < 0.001). Geographic location had no significant direct effect (path coefficient = 0.197, *p* > 0.05), but played a critical indirect regulatory role. Specifically, geographic location significantly influenced physicochemical parameters including temperature, total dissolved solids (TDS), dissolved oxygen (DO), and pH (path coefficient = −0.700, *p* < 0.001), which in turn shaped nutrient distribution (phosphate, silicate, ammonium, nitrate, and nitrite; total effect = 0.28). Notably, neither physicochemical parameters nor nutrients exerted significant independent direct effects on community structure (*p* > 0.05), indicating that their influences were embedded within the overall temporal successional process observed during the study period.

Redundancy analysis (RDA) further quantified the contribution of environmental factors to genus-level community structure (Figure 6C). The first two RDA axes together accounted for 50.21% of the observed community variation (RDA1: 45.11%; RDA2: 5.10%). Consistent with envfit permutation tests, temperature, silicate, and nitrate emerged as the key dominant drivers (red arrows, *p* < 0.001). January samples clustered in the negative region of RDA1, corresponding to higher silicate and nitrate concentrations and lower temperatures. In contrast, February samples shifted toward the positive region of RDA1, associated with elevated temperatures and ammonium accumulation.

A heatmap illustrating FDR-adjusted genus-level species–environment correlations clarified the distinct response patterns of key bacterial genera to environmental drivers (Figure 6D). Nitrate emerged as the most influential nutrient factor, with strong correlations to several dominant genera: it exhibited significant negative correlations with *Phenylobacterium*, *Ralstonia* (*p* < 0.01), and *Herbaspirillum* (*p* = 0.010), while maintaining a robust positive association with *Candidatus_Pelagibacter* (*r* = 0.628, *p* < 0.01). Silicate also exerted a regulatory effect, showing a significant positive correlation with *Polaribacter* (*p* < 0.05). Temperature, meanwhile, was significantly negatively correlated with *Roseibacillus* (*p* < 0.05). In contrast, other parameters including TDS, pH, DO, and nitrite showed no significant associations with any of the target bacterial genera following FDR correction (*p* > 0.05), indicating their limited direct influence on the observed taxonomic shifts.

## 4 Discussion

Our results demonstrated that the prokaryotic community in Maxwell Bay was dominated by the phyla *Proteobacteria* and *Bacteroidota*, with this compositional profile consistent with findings from other Antarctic marine regions (Kim et al., 2020; Cordone et al., 2022). Notably, significant community succession took place between January and February, with distinct changes in both alpha and beta diversity. Community composition shifted markedly: *Alphaproteobacteria* and *Flavobacteriia* (e.g., *Polaribacter* and *Sulfitobacter*) were dominant in January, while *Betaproteobacteria* (e.g., *Herbaspirillum* and *Ralstonia*) became predominant in February. This compositional turnover extended beyond simple species replacement, reflecting a potential transition in the community’s metabolic trajectory, specifically a shift from a “photo-heterotrophic” mode toward a lifestyle coupled to nitrogen cycling. Similar patterns of community turnover have been reported in other Antarctic investigations (Yergeau et al., 2007; Li et al., 2019), with temperature and nitrate identified as key drivers of these shifts. The consistent successional dynamics observed herein align with microbial adaptive strategies across distinct Antarctic habitats, further supporting coordinated community responses among polar microbes in the face of environmental change.

Phylogenetic null model-based analysis of community assembly mechanisms revealed a striking shift in the processes structuring bacterial communities: the January community was shaped by a balance between stochastic and deterministic processes, whereas homogeneous selection dominated the February community, accounting for 84.68% of the observed variation. As the austral summer advanced, increasingly stringent environmental selection pressures including rising temperatures and nutrient depletion strongly filtered for adapted taxa. Our results further reveal that the shift toward deterministic community assembly was mainly governed by discrete environmental variables. Specifically, temperature represented the dominant driver in January (*R^2^* = 0.062, *p* = 0.006), whereas silicate (*R^2^* = 0.11, *p* < 0.001) and nitrate (*R^2^* = 0.045, *p* = 0.02) acted as the primary controlling factors in February (Figures 5C–E). This transition substantially reduced the contribution of stochastic processes such as dispersal limitation and ecological drift, as evidenced by the pronounced downward shift of βNTI values to below the −2 threshold in February.

This finding offers a compelling illustration of environmental selection and aligns with successional patterns documented in microbial communities across diverse ecosystems. Numerous studies have shown that early-season or colonizing microbial communities are frequently structured by stochastic processes (Dini-Andreote et al., 2015), with deterministic processes gaining dominance as environmental pressure intensifies (Dini-Andreote et al., 2015; Doherty et al., 2020; Attia et al., 2022). Significant correlations between βNTI and specific environmental variables indicate that microbial community succession in this extreme polar environment is regulated by distinct niche-based selection, rather than a generalized response to broad-scale environmental change. Furthermore, this successional trajectory is supported by individual Mantel tests (Supplementary Table S2) and is consistent with findings from Arctic permafrost thaw, thus indicating a shared ecological response to thermal and nutrient stress across both polar regions (Doherty et al., 2020).

Through integrated multivariate statistical analyses including PLS-PM, RDA, and correlation analysis, this study further pinpointed the key environmental factors driving this community succession. Temporal variation proved to be the overarching primary driver, functioning as a “master switch” for community shifts; its effects were largely mediated by dynamic changes in environmental conditions, specifically temperature and nutrient dynamics, which collectively shaped the environmental selection of bacterial communities. Genus-level correlation heatmaps further clarified which taxa are regulated by these factors, such as the negative correlation between *Herbaspirillum* and nitrate. Global research on marine microorganisms has also found that temperature is the primary environmental factor influencing microbial community composition, outweighing the effects of geographical distance or other parameters (Sunagawa et al., 2015). As the most fundamental physical factor shaping microbial metabolic rates and community structure, temperature changes directly regulate microbial physiological activities, in turn affecting their abundance and diversity (Aalto et al., 2022; Liang et al., 2022). Meanwhile, nitrate, a critical nitrogen source, exerts a direct influence on microbial growth and community composition, particularly for phytoplankton and nitrogen-fixing bacteria. Studies have shown that nitrate concentrations are significantly positively correlated with the abundance of certain carbon-sequestering taxa (Ji, 2016). Furthermore, research on climate-driven microbial succession has shown that temperature fluctuations and nutrient dynamics jointly regulate microbial taxonomic turnover, which is coupled to shifts in associated biogeochemical processes (Larkin et al., 2025). Thus, the results of this Antarctic study reinforce the global pattern wherein temperature and nutrients co-drive microbial succession, underscoring the central role of multi-factor synergy in polar microbial ecology. It is important to note, however, that these factors do not act in isolation; they interact with other environmental variables (e.g., salinity, dissolved oxygen, phosphate) to comprehensively shape marine microbial habitats (Aalto et al., 2022; Liang et al., 2022; Wang et al., 2024).

## 5 Conclusion

This study demonstrates a pronounced temporal succession in the bacterial community structure within Maxwell Bay’s surface waters over the austral summer. A marked shift occurred at the genus level: the January community was characterized by the dominance of *Polaribacter* and *Sulfitobacter*, whereas February saw a prevalence of *Herbaspirillum* and *Ralstonia*. Since these late-summer dominant genera are typically associated with nitrogen metabolism, this compositional turnover indicates a potential transition in the community’s metabolic trajectory toward enhanced nitrogen cycling. Concurrent with this taxonomic turnover was a fundamental shift in community assembly mechanisms: early-summer (January) assembly was shaped by a balance of stochastic and deterministic processes, while late-summer assembly was predominantly governed by deterministic homogeneous selection (84.68%). Our analysis further clarifies that this transition reflects intensified environmental selection driven by individual environmental factors, particularly increasing water temperature and the fluctuations in nitrate and silicate concentrations. These findings highlight the sensitivity of polar microbial communities to temporal environmental change and provide critical insights for anticipating ecosystem-level responses to ongoing climate change.

Future research should adopt high-resolution temporal sampling and advanced genomic tools, such as metagenomics, to further investigate the short-term dynamics, succession patterns, and underlying ecological mechanisms of Antarctic summer microbiomes. Such investigations are crucial for accurately predicting the responses of polar ecosystems to climate change.

## Data Availability

The data presented in this study are publicly available. The data can be found here: https://ngdc.cncb.ac.cn/gsa, accession number CRA034201 (Bao et al., 2024).

## References

[ref1] Aalto N. J. Schweitzer H. D. Krsmanovic S. Campbell K. Bernstein H. C. (2022). Diversity and selection of surface marine microbiomes in the Atlantic-influenced Arctic. Front. Microbiol. 13:892634. doi: 10.3389/fmicb.2022.892634, 35910621 PMC9329088

[ref2] Amaro E. Padeiro A. De Ferro A. M. Mota A. M. Leppe M. Verkulich S. . (2015). Assessing trace element contamination in Fildes peninsula (King George Island) and Ardley Island, Antarctic. Mar. Pollut. Bull. 97, 523–527. doi: 10.1016/j.marpolbul.2015.05.018, 25982820

[ref3] Attia S. Russel J. Mortensen M. S. Madsen J. S. Sorensen S. J. (2022). Unexpected diversity among small-scale sample replicates of defined plant root compartments. ISME J. 16, 997–1003. doi: 10.1038/s41396-021-01094-7, 34759302 PMC8940884

[ref4] Bao Y. M. Zhang Z. Zhao W. M. Xiao J. F. Song S. H. He S. M. . (2024). Database resources of the National Genomics Data Center, China National Center for bioinformation in 2025. Nucleic Acids Res. 53, D30–D44. doi: 10.1093/nar/gkae978, 39530327 PMC11701749

[ref5] Barbraud C. Rolland V. Jenouvrier S. Nevoux M. Delord K. Weimerskirch H. (2012). Effects of climate change and fisheries bycatch on Southern Ocean seabirds: a review. Mar. Ecol. Prog. Ser. 454, 285–307. doi: 10.3354/meps09616

[ref6] Bello C. Suarez W. Lavado-Casimiro W. (2022). Trends and space–time patterns of near-surface temperatures on Maxwell Bay, King George Island, Antarctica. Int. J. Climatol. 42, 7426–7442. doi: 10.1002/joc.7661

[ref7] Bendia A. G. Moreira J. C. F. Ferreira J. C. N. Romano R. G. Ferreira I. G. C. Franco D. C. . (2023). Insights into Antarctic microbiomes: diversity patterns for terrestrial and marine habitats. An. Acad. Bras. Cienc. 95:e20211442. doi: 10.1590/0001-3765202320211442, 37820122

[ref8] Bendschneider K. Robinson R. J. (1952). A new spectrophotometric method for the determination of nitrite in sea water. J. Mar. Res. 11, 87–96.

[ref9] Caporaso J. G. Lauber C. L. Walters W. A. Berg-Lyons D. Lozupone C. A. Turnbaugh P. J. . (2011). Global patterns of 16S rRNA diversity at a depth of millions of sequences per sample. Proc. Natl. Acad. Sci. USA 108 Suppl 1, 4516–4522. doi: 10.1073/pnas.1000080107, 20534432 PMC3063599

[ref10] Cavicchioli R. (2015). Microbial ecology of Antarctic aquatic systems. Nat. Rev. Microbiol. 13, 691–706. doi: 10.1038/nrmicro3549, 26456925

[ref11] Convey P. Bindschadler R. di Prisco G. Fahrbach E. Gutt J. Hodgson D. A. . (2009). Antarctic climate change and the environment. Antarct. Sci. 21, 541–563. doi: 10.1017/s0954102009990642

[ref12] Cordone A. D'Errico G. Magliulo M. Bolinesi F. Selci M. Basili M. . (2022). Bacterioplankton diversity and distribution in relation to phytoplankton community structure in the Ross Sea surface waters. Front. Microbiol. 13:722900. doi: 10.3389/fmicb.2022.722900, 35154048 PMC8828583

[ref13] Davidson A. T. McKinlay J. Westwood K. Thomson P. G. van den Enden R. de Salas M. . (2016). Enhanced CO2 concentrations change the structure of Antarctic marine microbial communities. Mar. Ecol. Prog. Ser. 552, 93–113. doi: 10.3354/meps11742

[ref14] Dini-Andreote F. Stegen J. C. Van Elsas J. D. Salles J. F. O. (2015). Disentangling mechanisms that mediate the balance between stochastic and deterministic processes in microbial succession. Proc. Natl. Acad. Sci. USA 112, E1326–E1332. doi: 10.1073/pnas.1414261112, 25733885 PMC4371938

[ref15] Doherty S. J. Barbato R. A. Grandy A. S. Thomas W. K. Monteux S. Dorrepaal E. . (2020). The transition from stochastic to deterministic bacterial community assembly during permafrost thaw succession. Front. Microbiol. 11:596589. doi: 10.3389/fmicb.2020.596589, 33281795 PMC7691490

[ref16] Edgar R. C. (2013). UPARSE: highly accurate OTU sequences from microbial amplicon reads. Nat. Methods 10, 996–998. doi: 10.1038/nmeth.2604 23955772

[ref17] Edgar R. (2016) SINTAX: a simple non-Bayesian taxonomy classifier for 16S and ITS sequences [Epubh ahead of preprint]. doi: 10.1101/074161

[ref18] Faria L. C. Raut Y. McNichol J. Williams N. L. R. Fuhrman J. A. Signori C. N. (2025) A multidomain lens on the temporal dynamics of surface microbial communities in the Southern Ocean (2013-2019) [Epubh ahead of preprint]. doi: 10.1101/2025.03.27.645779 41967216

[ref19] Goldenberg-Vilar A. Moran-Luis M. Vieites D. R. alvarez-Martinez J. M. Silio A. Mony C. . (2025). Biogeographical distribution of river microbial communities in Atlantic catchments. Environ. Microbiol. Rep. 17:e70065. doi: 10.1111/1758-2229.70065, 39776267 PMC11707552

[ref20] Henley S. F. Cavan E. L. Fawcett S. E. Kerr R. Monteiro T. Sherrell R. M. . (2020). Changing biogeochemistry of the Southern Ocean and its ecosystem implications. Front. Mar. Sci. 7:581. doi: 10.3389/fmars.2020.00581

[ref21] Iqbal S. Begum F. Nguchu B. A. Claver U. P. Shaw P. (2025). The invisible architects: microbial communities and their transformative role in soil health and global climate changes. Environ. Microbiome. 20:36. doi: 10.1186/s40793-025-00694-6, 40133952 PMC11938724

[ref22] Isla E. (2022). The southern ocean:our best opportunity? Arq. Ciênc. Mar. 55, 180–190. doi: 10.32360/acmar.v55iespecial.78406

[ref23] Jarnikova T. Le Quere C. Rumbold S. Jones C. (2025). Decreasing importance of carbon-climate feedbacks in the Southern Ocean in a warming climate. Sci. Adv. 11:eadr3589. doi: 10.1126/sciadv.adr3589, 40378228 PMC12083540

[ref24] Ji F. Y. (2016). Microbial Community Composition of Liaohe in Seawater and Pearl river Estuary in Sediment [Master's Degree thesis]. Dalian (China): Dalian Ocean University.

[ref25] Kim S. Kim J.-H. Lim J.-H. Jeong J.-H. Heo J.-M. Kim I.-N. (2020). Distribution and control of bacterial community composition in Marian cove surface waters, King George Island, Antarctica during the summer of 2018. Microorganisms 8:1115. doi: 10.3390/microorganisms8081115, 32722258 PMC7464920

[ref26] Larkin A. A. Brock M. L. Fagan A. J. Moreno A. R. Gerace S. D. Lees L. E. . (2025). Climate-driven succession in marine microbiome biodiversity and biogeochemical function. Nat. Commun. 16:3926. doi: 10.1038/s41467-025-59382-1, 40280934 PMC12032349

[ref27] Li Y. Cha Q. Q. Dang Y. R. Chen X.-L. Wang M. McMinn A. . (2019). Reconstruction of the functional ecosystem in the high light, low temperature union glacier region, Antarctica. Front. Microbiol. 10:2408. doi: 10.3389/fmicb.2019.02408, 31681251 PMC6813960

[ref28] Liang Z. R. Jia R. J. Sun T. Q. Wang W. J. Wang C. Lu X. P. (2022). Insights into the spatio-temporal composition, diversity and function of bacterial communities in seawater from a typical laver farm. Front. Mar. Sci. 9:1056199. doi: 10.3389/fmars.2022.1056199

[ref29] Liu Q. Jiang Y. (2020). Application of microbial network analysis to discriminate environmental heterogeneity in Fildes peninsula, Antarctica. Mar. Pollut. Bull. 156:111244. doi: 10.1016/j.marpolbul.2020.111244, 32510386

[ref30] Luo W. Li H. R. Gao S. Q. Yu Y. Lin L. Zeng Y. X. (2016). Molecular diversity of microbial eukaryotes in sea water from Fildes peninsula, King George Island, Antarctica. Polar Biol. 39, 605–616. doi: 10.1007/s00300-015-1815-8

[ref31] Luria C. M. Amaral-Zettler L. A. Ducklow H. W. Repeta D. J. Rhyne A. L. Rich J. J. (2017). Seasonal shifts in bacterial community responses to phytoplankton-derived dissolved organic matter in the Western Antarctic peninsula. Front. Microbiol. 8:2117. doi: 10.3389/fmicb.2017.02117, 29163409 PMC5675858

[ref32] Mora C. Spirandelli D. Franklin E. C. Lynham J. Kantar M. B. Miles W. . (2018). Broad threat to humanity from cumulative climate hazards intensified by greenhouse gas emissions. Nat. Clim. Chang. 8, 1062–1071. doi: 10.1038/s41558-018-0315-6

[ref33] Morris A. Riley J. (1963). The determination of nitrate in sea water. Anal. Chim. Acta 29, 272–279. doi: 10.1016/S0003-2670(00)88614-6

[ref34] Murphy E. J. Johnston N. M. Hofmann E. E. Phillips R. A. Jackson J. A. Constable A. J. . (2021). Global connectivity of Southern Ocean ecosystems. Front. Ecol. Evol. 9:624451. doi: 10.3389/fevo.2021.624451

[ref35] Murphy J. Riley J. P. (1962). A modified single solution method for the determination of phosphate in natural waters. Anal. Chim. Acta 27, 678–681. doi: 10.1016/s0003-2670(00)88444-5

[ref36] Nack K. Boutin J. Swart S. Duplessis M. Merlivat L. Beaumont L. . (2025). Anomalous summertime CO2 sink in the subpolar Southern Ocean promoted by early 2021 sea ice retreat. Biogeosciences 22, 1947–1968. doi: 10.5194/bg-22-1947-2025

[ref37] Ong E. Q. Y. England M. H. Doddridge E. Constantinou N. C. (2025). Transient Antarctic slope current response to climate change including meltwater. Geophys. Res. Lett. 52:e2024GL113983. doi: 10.1029/2024gl113983

[ref38] Piedade G. J. Schoen M. E. Lood C. Fofanov M. V. Wesdorp E. M. Biggs T. E. G. . (2024). Seasonal dynamics and diversity of Antarctic marine viruses reveal a novel viral seascape. Nat. Commun. 15:9192. doi: 10.1038/s41467-024-53317-y 39448562 PMC11502894

[ref39] Quast C. Pruesse E. Yilmaz P. Gerken J. Schweer T. Yarza P. . (2012). The SILVA ribosomal RNA gene database project: improved data processing and web-based tools. Nucleic Acids Res. 41, D590–D596. doi: 10.1093/nar/gks1219, 23193283 PMC3531112

[ref40] Rego A. Sousa A. G. G. Santos J. P. Pascoal F. Magalhes C. (2020). Diversity of bacterial biosynthetic genes in maritime Antarctica. Microorganisms 8:279. doi: 10.3390/microorganisms8020279, 32085500 PMC7074882

[ref41] Rozema P. D. Biggs T. Sprong P. A. A. Buma A. G. J. Venables H. J. Evans C. . (2017). Summer microbial community composition governed by upper-ocean stratification and nutrient availability in northern Marguerite Bay, Antarctica. Deep Sea Res. Part II Top. Stud. Oceanogr. 139, 151–166. doi: 10.1016/j.dsr2.2016.11.016

[ref42] Siman-Tov I. (2022). Metagenomic Analysis of Microbial 18s Eukaryotes Communities and Environmental Factors in the Western Antarctic Peninsula Waters During Austral Summers [master's thesis]. San Jose (CA): San Jose State University.

[ref43] Steinberg D. K. Landry M. R. Annual R. (2017). Zooplankton and the ocean carbon cycle. Annu. Rev. Mar. Sci. 9, 413–444. doi: 10.1146/annurev-marine-010814-015924 27814033

[ref44] Sunagawa S. Coelho L. P. Chaffron S. Kultima J. R. Labadie K. Salazar G. . (2015). Structure and function of the global ocean microbiome. Science 348:1261359. doi: 10.1126/science.1261359, 25999513

[ref45] Thomsen J. Johnson K. S. Petty R. L. (1983). Determination of reactive silicate in seawater by flow injection analysis. Anal. Chem. 55, 2378–2382. doi: 10.1021/ac00264a039

[ref46] Wang Z. Gao Z. W. Yu Y. Li H. R. Luo W. Ji Z. Q. . (2024). New insights into the structure and function of microbial communities in Maxwell Bay, Antarctica. Front. Microbiol. 15:1463144. doi: 10.3389/fmicb.2024.1463144, 39296290 PMC11408308

[ref47] Wilkins D. Yau S. Williams T. J. Allen M. A. Brown M. V. DeMaere M. Z. . (2013). Key microbial drivers in Antarctic aquatic environments. FEMS Microbiol. Rev. 37, 303–335. doi: 10.1111/1574-6976.12007, 23062173

[ref48] Yergeau E. Bokhorst S. Huiskes A. H. L. Boschker H. T. S. Aerts R. Kowalchuk G. A. (2007). Size and structure of bacterial, fungal and nematode communities along an Antarctic environmental gradient. FEMS Microbiol. Ecol. 59, 436–451. doi: 10.1111/j.1574-6941.2006.00200.x, 16978243

[ref49] Zeng Y. X. Yu Y. Qiao Z. Y. Jin H. Y. Li H. R. (2014). Diversity of bacterioplankton in coastal seawaters of Fildes peninsula, King George Island, Antarctica. Arch. Microbiol. 196, 137–147. doi: 10.1007/s00203-013-0950-2, 24408126

[ref50] Zhang S. S. Chen X. Jin E. H. Wang A. K. Chen T. T. Zhang X. L. . (2025). The GSA family in 2025: a broadened sharing platform for multi-omics and multimodal data. Genomics Proteomics Bioinformatics 23:qzaf072. doi: 10.1093/gpbjnl/qzaf072, 40857552 PMC12451262

[ref51] Zhang Y. H. Hu Y. Q. Zeng Y. X. Hu T. Han W. Du Y. . (2025). Bacterial community composition and function in different habitats in Antarctic Fildes region revealed by high-throughput sequencing. Front. Microbiol. 16:1524681. doi: 10.3389/fmicb.2025.1524681 40589579, 40589579 PMC12206884

[ref52] Zhang J. J. Kobert K. Flouri T. Stamatakis A. (2014). PEAR: a fast and accurate Illumina paired-end reAd mergeR. Bioinformatics 30, 614–620. doi: 10.1093/bioinformatics/btt593, 24142950 PMC3933873

[ref53] Zhang Y. Lu L. Chang X. Jiang F. Gao X. Yao Y. . (2018). Small-scale soil microbial community heterogeneity linked to landform historical events on King George Island, maritime Antarctica. Front. Microbiol. 9:3065. doi: 10.3389/fmicb.2018.03065, 30619151 PMC6296293

