# Supplementary material for: Temporal succession and assembly of marine bacterial communities in Maxwell Bay, Antarctica during summer
Source: Front Microbiol. 2026 Mar 19;17:1748960. doi: 10.3389/fmicb.2026.1748960 (PMC13044028; doi:10.3389/fmicb.2026.1748960)
Supplement: Supplementary file 1 [file Table_1.DOCX]

| Environmental Parameters | Jan | Feb |
| --- | --- | --- |
| Temperature (℃) | 1.71±0.28 | 1.82±0.12 |
| Total Dissolved Solids (g/L) | 53.58±0.42 | 53.31±0.60 |
| pH | 7.83±0.03 | 7.83±0.03 |
| Dissolved oxygen (mg/L) | 13.50±0.53 | 13.40±0.69 |
| Phosphate (µmol/L) | 2.09±0.42 | 1.67±0.69 |
| Silicate (µmol/L) | 44.41±2.88 | 37.94±4.94 |
| Ammonium (µmol/L) | 5.33±1.67 | 5.78±1.71 |
| Nitrate (µmol/L) | 25.30±5.70 | 18.03±5.21 |
| Nitrite (µmol/L) | 0.18±0.04 | 0.17±0.03 |

Table S1**.** Statistical characteristics of environmental factors in January and February.
