# Supplementary material for: Temporal succession and assembly of marine bacterial communities in Maxwell Bay, Antarctica during summer
Source: Front Microbiol. 2026 Mar 19;17:1748960. doi: 10.3389/fmicb.2026.1748960 (PMC13044028; doi:10.3389/fmicb.2026.1748960)
Supplement: Supplementary file 2 [file Table_2.DOCX]

| Month | Factor | Mantel_*r* | Mantel_*p* |
| --- | --- | --- | --- |
| Feb | Silicate | 0.2992 | 0.012 |
| Feb | Nitrate | 0.164 | 0.042 |
| Feb | pH | 0.2093 | 0.062 |
| Feb | Temperature | 0.2001 | 0.089 |
| Feb | Ammonium | 0.208 | 0.089 |
| Feb | Nitrite | 0.1834 | 0.097 |
| Feb | TDS | 0.0107 | 0.489 |
| Feb | DO | -0.0577 | 0.714 |
| Feb | Phosphate | -0.411 | 0.99 |
| Jan | Temperature | 0.2416 | 0.006 |
| Jan | Phosphate | 0.1067 | 0.238 |
| Jan | Ammonium | 0.0724 | 0.321 |
| Jan | Nitrate | 0.071 | 0.326 |
| Jan | Nitrite | 0.0462 | 0.424 |
| Jan | pH | 0.0109 | 0.458 |
| Jan | TDS | 0.0241 | 0.472 |
| Jan | DO | -0.0261 | 0.601 |
| Jan | Silicate | -0.0866 | 0.77 |

Table S2. Mantel test results for the correlations between environmental factors and *β*NTI in January and February.
