# Supplementary material for: Temporal succession and assembly of marine bacterial communities in Maxwell Bay, Antarctica during summer
Source: Front Microbiol. 2026 Mar 19;17:1748960. doi: 10.3389/fmicb.2026.1748960 (PMC13044028; doi:10.3389/fmicb.2026.1748960)
Supplement: Supplementary file 3 [file Table_3.DOCX]

| Month | Factor | *R*_squared | *P*_value | Slope |
| --- | --- | --- | --- | --- |
| Feb | Phosphate | 0.131 | 0 | -0.3651 |
| Feb | Silicate | 0.1089 | 2.00E-04 | 0.3583 |
| Feb | pH | 0.0561 | 0.0092 | 0.2554 |
| Feb | Temperature | 0.0474 | 0.0169 | 0.2185 |
| Feb | Nitrite | 0.0467 | 0.0177 | 0.2371 |
| Feb | Nitrate | 0.0448 | 0.0203 | 0.2384 |
| Feb | Ammonium | 0.0397 | 0.0291 | 0.1935 |
| Feb | TDS | 0.0015 | 0.6723 | 0.0384 |
| Feb | DO | 0.0015 | 0.6795 | -0.0438 |
| Jan | Temperature | 0.0622 | 0.006 | 0.2141 |
| Jan | Silicate | 0.0139 | 0.2 | -0.1004 |
| Jan | Phosphate | 0.0095 | 0.2895 | 0.0656 |
| Jan | Nitrate | 0.0084 | 0.3181 | 0.0656 |
| Jan | Nitrite | 0.0062 | 0.392 | 0.0535 |
| Jan | Ammonium | 0.0054 | 0.4251 | 0.0534 |
| Jan | TDS | 0.0034 | 0.5256 | 0.0455 |
| Jan | pH | 4.00E-04 | 0.8288 | -0.0162 |
| Jan | DO | 3.00E-04 | 0.8572 | -0.0142 |

Table S3. Linear regression analysis of the effects of environmental factors on *β*NTI in January and February.
