# Supplementary figures and images for: Temporal succession and assembly of marine bacterial communities in Maxwell Bay, Antarctica during summer
Source: Front Microbiol. 2026 Mar 19;17:1748960. doi: 10.3389/fmicb.2026.1748960 (PMC13044028; doi:10.3389/fmicb.2026.1748960)

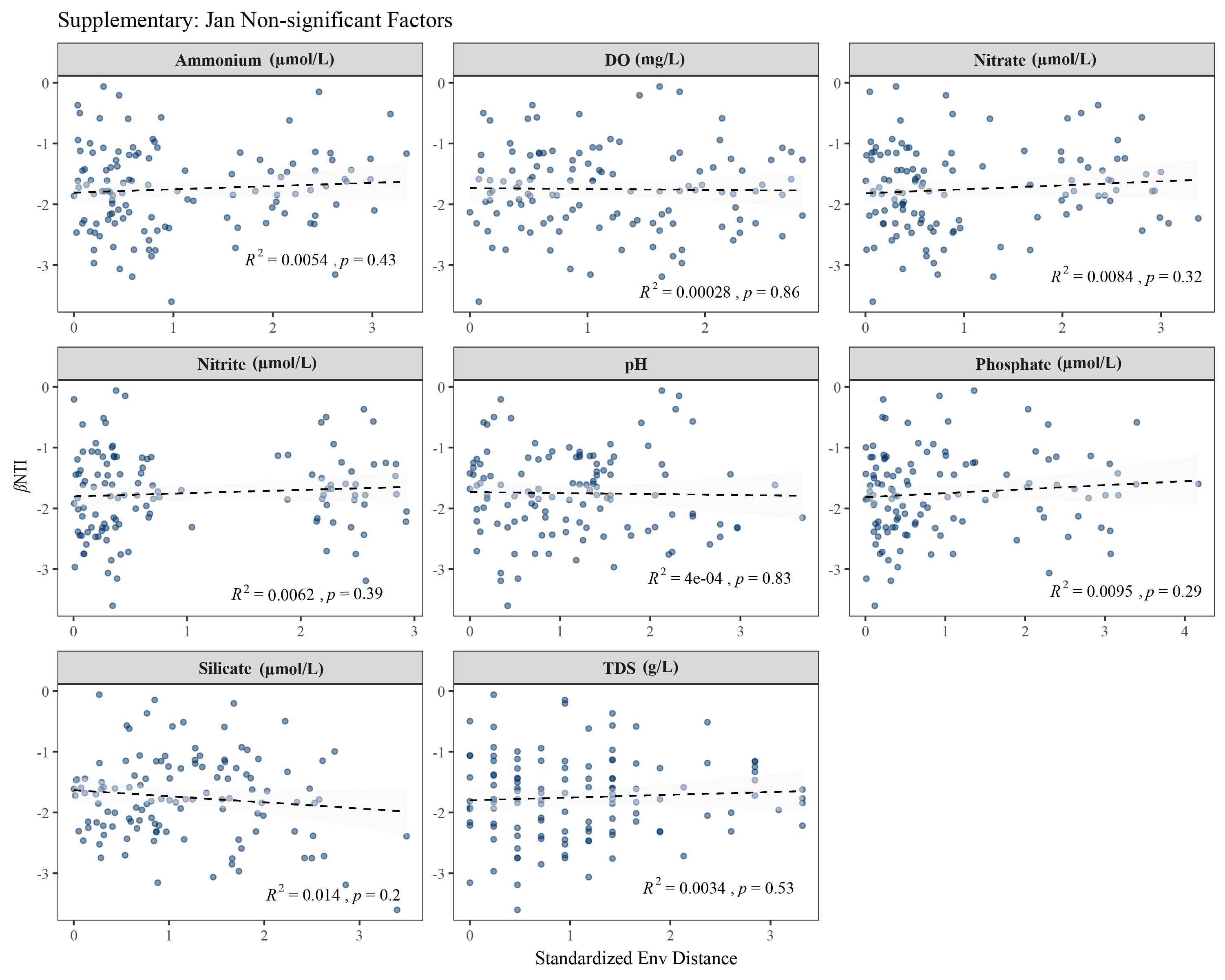

Supplement: Supplementary file 5 [file Image_2.JPEG]

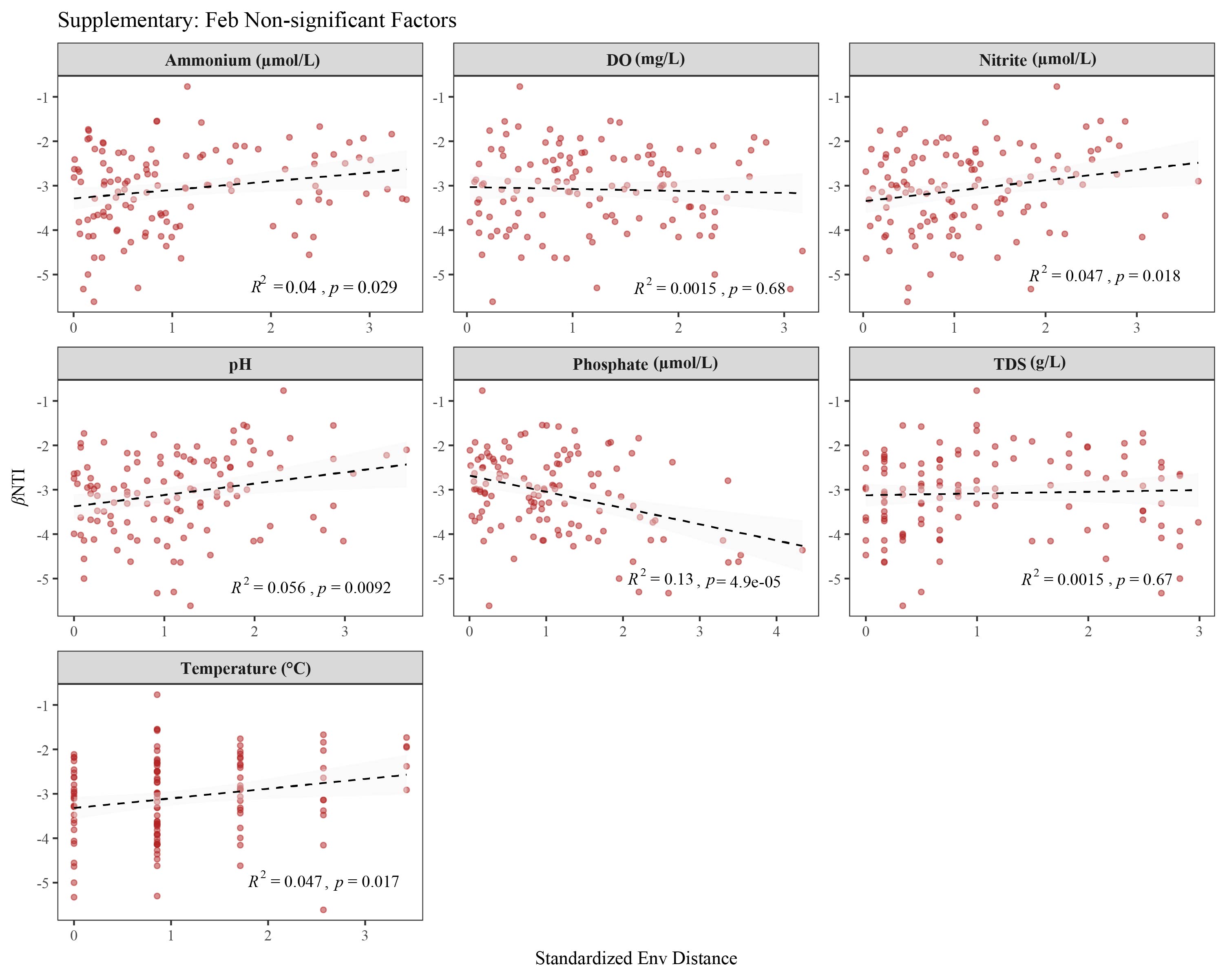

Supplement: Supplementary file 6 [file Image_3.JPEG]
